# Anxiety Behavior in Pigs (*Sus scrofa*) Decreases Through Affiliation and May Anticipate Threat

**DOI:** 10.3389/fvets.2021.630164

**Published:** 2021-02-16

**Authors:** Ivan Norscia, Edoardo Collarini, Giada Cordoni

**Affiliations:** Department of Life Sciences and Systems Biology, University of Torino, Turin, Italy

**Keywords:** animal emotion, comparative psychology, stress, evolution of emotional behavior, extensive farming, swine, domestication, pig welfare

## Abstract

Anxiety is a physio-psychological state anticipating an imminent threat. In social mammals it is behaviorally expressed *via* displacement activities and buffered *via* affiliation. Anxiety research on domestic pigs (*Sus scrofa*) has mostly focused on abnormal/stereotypic behavior associated with intensive farming. We investigated how anxiety is expressed and modulated in semi-free ranging pigs, in natural habitats. Owing to pigs' socio-cognitive complexity, we posited that displacement activities, if such, would increase after a (stressful) intra-group aggression (Prediction 1), be reduced by affiliation (Prediction 2) and influenced by individual/contextual factors (Prediction 3). From 224 videos recorded on adult individuals (Mean ± SD/subject: 4.84 ± 1.85 h) at the “Ethical Farm Parva Domus” (Turin, Italy), we extracted possible displacement activities (*vacuum-chewing, scratching/body-rubbing, head/body-shaking*, and *yawning*) in four 3-min conditions: before (BA) and after aggression events, in the absence (AA) or presence (AP) of post-aggression affiliation, and a matched-control (no event; MC). We conducted a minute-by-minute analysis in AE/AA and assessed the effect of subjects' involvement in a conflict (aggressor, aggression's recipient, bystander). All activities were higher in AA than in BA condition—thus being anxiety markers—and all of them decreased to baseline levels in AP, faster compared to AE. Hence, anxiety behavior in pigs was socially buffered. Intriguingly, anxiety behavior was expressed significantly more by bystanders than opponents, which suggests that pigs may be able to anticipate imminent threats. By highlighting how anxiety is managed under extensive farming, this study contributes to the understanding of pig welfare and biology.

## Introduction

In its broader definition, anxiety is an affective state that in both humans and other mammals is characterized by tension and/or agitation and is often behaviorally expressed in association with a physiological stress response (1–5). In mammals, including *Homo sapiens*, genetic predisposition, early life experiences, and overcrowding conditions can increase the propensity to develop pathological anxiety and/or anxiety-related stereotypic behavior (6–14). However, non-pathological anxiety is commonly present in a mammal's life as a psychological, physiological, and behavioral response that helps the subjects to deal with unexpected or challenging situations (15). Probably owing to this basic function, convergence in some anxiety related behaviors has been observed between humans and non-human mammals (16).

Intra-group aggression is a major source of anxiety in social mammals (17). Indeed, agonistic encounters carry the risk of physical harm for individuals and can be disruptive for social groups, unless they are resolved *via* conflict management (18). The anxiety response to aggression can not only be present but may also vary depending on the role played by individuals in the conflict [e.g., pigs (19); rats, *Rattus norvegicus* (20); various non-human primate species (17, 21–25)]. For example, the aggressor can show lower levels of anxiety than the aggression's recipient and the bystanders not involved in the conflict can also show an increase in anxiety [e.g., rats (26); *Lemur catta* (27); *Papio anubis* (28); *Papio hamadryas* (29, 30)]. The behavioral expression of anxiety in agonistic contexts is therefore necessary to ensure effective communication between individuals within a society and allow the enactment of social buffering measures (20, 31, 32).

Besides the behaviors that in animals are generally associated with the stress response to fear [e.g., urinating, defecating, escape (33)], there are other displacement activities more strictly linked to anxiety. Some are present in a variety of mammalian species and are therefore interesting to investigate in the light of future comparative studies. For example, depending on the severity of the stressor eliciting a response, self-scratching (hereafter scratching), yawning, vacuum behavior [*sensu* (34)], and head/body shaking or trembling, can be associated with chronic and/or acute anxiety in both human and non-human primates (4, 35–39), and in other mammals, including domestic animals (40–44). From the physiological perspective, such behaviors appear to be linked to the hormonal cascade that underlies the stress response, mediated by cortisol and whose intensity depends on the stressor (40, 45–47). Scratching, in particular, is performed in response to the itch sensation mediated by cortisol (48, 49) and can lead to the itch-scratch cycle (50).

In social mammals, anxiety can be buffered by affinitive interactions between subjects, such as closeness, body contact, and allogrooming (32). This social buffering mechanism has been observed in both primates [humans (51); non-human primates (52, 53)], and non-primate mammals [rats (54); guinea pigs, *Cavia porcellus* (55); prairie voles, *Microtus ochrogaster* (56); pigs (57)]. Indeed, affinitive contacts are known to reduce anxiety behavior in rodents (58), even though primates have been the most investigated group in this respect (24). From strepsirrhines to apes, displacement behavior has been found to be reduced after grooming (27), play (59), and post-conflict affinitive contacts with a former opponent or another group mate [(60–63); but see (64)]. Studies of diverse mammalian species, including humans, suggest that affiliation may cause the reduction of stress related anxiety *via* the activation of oxytocin, progesteron and/or endorphines mediated responses (65–67). In an experimental setting, domestic pigs have been found to prefer staying close to familiar rather than unfamiliar subjects after a stressful conflict (68).

Although commonly known and available as study species, the domestic pig (*Sus scrofa*) has received relatively little attention with respect to its social behavior in extensive, naturalistic farming conditions because the majority of clinical ethology research has focused on behavioral alterations related to intensive, industrial food production chain. More specifically, stress and anxiety in the domestic pig *Sus scrofa* have been broadly investigated with respect to intensive farming [for a review (11)]. However, to our knowledge no ethological study has so far quantitatively demonstrated the association of certain behaviors of *Sus scrofa* with the non-stereotipic display of anxiety and social buffering under naturalistic conditions. Yet, the domestic pig, *Sus scrofa*, is an excellent species to investigate emotional expression and modulation potentials in non-human social mammals, owing to its complex sociality, cognition and psychology (69). Moreover, it seems that domestic species display more anxiety-like and less risk-taking and exploratory behavior than wild forms, as a result of the domestication process (70).

The aim of this study was to investigate the behavioral manifestation of transient anxiety and its management in *Sus scrofa*, by adopting the same approach used for non-domestic species, in an ecologically sound context. In this condition—as it also occurs in other settings (e.g., open field tests)—the subjects can express their behavioral repertoire without the distortion caused by confinement and overcrowding. This issue is relevant not only to the understanding of pig emotional expression and modulation, but also to gain insights into pig welfare, considering the growing interest toward extensive and environmentally sustainable farming. To achieve this aim, we examined a suite of behaviors associated with chronic conditions in pigs or to chronic/acute stress in other mammals, and checked whether they could also be connected to transient anxiety in pigs. In particular, we measured the fluctuation of these behaviors in semi-free ranging pigs, living in a 13 ha habitat of natural mixed wood and grassland.

Based on the framework presented above, we expected that in *Sus scrofa*: (i) body shaking, vacuum chewing, yawning, and the behavior associated with the itch-scratch cycle (scratching/rubbing)—if linked to non-pathological anxiety—would increase after an aggressive event (Prediction 1); (ii) such behavior—if socially buffered—would be reduced in the subjects after affiliation with group mates (Prediction 2); (iii) the occurrence of anxiety related behavior—if modulated according to the risk faced by the subjects—could vary according to the role the subjects played in the aggression (i.e., aggressor, recipient or bystander) (Prediction 3).

## Materials and Methods

### Study Group and Site

This study was conducted from June to November 2018 on a group of free-ranging domestic pigs (*Sus scrofa*) at the Ethical Farm “Parva Domus” located at Cavagnolo, Turin (Italy). The animals could freely move and forage in a natural habitat, including both grassland and woodland, within a fenced area of 13 ha. The 104 adult subjects (7–22 months old) lived in the same group and included 54 males and 50 females of three mixed breeds: Parma Black, Landrace, and Piedmont Black. The animals had been together from 3 to 14 months and no subject showed stereotypic behavior during the study (e.g., repeated and abnormal behaviors in absence of any perturbing event). Owing to controlled reproduction, kinship varied from second cousins to full siblings. No parental relationship was present in the group. The males were castrated *via* the removal of testes within their first days of life, whereas females were potentially reproductive but reproductive males was kept in separate enclosures. Four feeding spots were available in the area where the animals were provided with food (Ciclo Unico P, SILDAMIN®) every day from 8:30 to 10:30 and water was available *ad libitum*. The subjects could supplement their food intake with roots, leaves or fruits naturally available in the environment. In order to allow the individual recognition of all subjects, the pigs were marked with spray Raidex^©^ for livestock. Each individual had a unique marking that was renewed every 4–7 days, depending on weather conditions. All subjects were well-habituated to human presence. Due to a low culling rate (usually one individual per week), the subjects included in this study were available for the whole data collection period.

### Observational Data, Operational Definitions, and Video Analysis

Videos were collected on the study animals on a daily basis (except in case of heavy rain), from 06:30 a.m. to 05:00 p.m. HD video records were collected by 2–3 operators each day—from different angles—to ensure broader or different visual ranges—using Panasonic HC-V380/V180 and Sony HDR-PJ240E video recorders. In total, 224 videos were collected, including one or more individuals, corresponding to 42.67 h of video observation (Mean ± SD/subject: 4.84 ± 1.85 h). From the videos we extracted 168 aggressive events and the occurrence of behaviors was recorded in 3 min blocks in different conditions before and after each event. From the videos we also recorded the occurrence of the same behaviors under the control condition (MC). All the conditions are described below.

The video-analysis started after a training phase with both supervisors (IN, GC), when interobserver reliability scores measured *via* Cohen's k reached 0.81. The inter-observer reliability between video coders was calculated using the R function “cohen.cappa” and libraries “irr” and “psych” (R version 3.5.3). The video-analyses were carried out *via* freeware VLC 3.0.6 and extension Jump-to-Time.

We considered as stressful events the aggressive encounters resulting in physical contact between opponents [aggressive behaviors are described in Table 1 (71)].

**Table 1:** Affinitive, aggressive, and (possible) anxiety behaviors considered in this study [integrated or modified from Bolhuis et al. (71), Petersen et al. (72), and Stolba and Wood Gush (73), Giersing and Andersson, (74); Jensen, (75); Sekiguchi and Koketsu, (76)].

**Behavioral pattern**	**Description**
**AFFINITIVE BEHAVIORS** (Figure 1)	
Rest in contact	Two subjects sit or lay in contact with one another
Social touching	A subject touches another with a paw or other body parts, except nose/head
Nose-body contact	A subject makes contact with another with its nose (*via* pushing or touching). For the purpose of this study, this also includes when the subjects touch each other's nose
Head-over	A subject puts its head above the back of another individual, followed by rest in contact or body contact
Nosing-body	Two subjects sniff each other on head, genitals, nose, and/or other body parts
**AGGRESSIVE BEHAVIORS**	
Aggressive lifting	A subject attempts to displace another by lifting or levering it with snout or head
Aggressive biting	A subject opens its mouth and close its teeth tight on another subject's small piece of flesh, including tail
Aggressive mounting	A subject force another individual to move away by rising upon the rear of another subject
Aggressive kicking	A subjects projects of one or both hind limbs toward another subject, striking it
Aggressive pushing	A subject presses its head, neck, shoulder, or body against another subject, causing the other individual to move.
Aggressive chasing	A subject pursues another subject, which flees
Aggressive head-knocking	A subject lurches or jerks its head hitting another subject
Fighting	Two subjects mutually push one another in a head to head orientation. The pattern often involve body-to-body rotation and can include aggressive mounting, lifting, biting, attempt biting, kicking, chasing, pushing, head knocking, high pitched vocalization, with no interruption lasting more than 10 s.
**DISPLACEMENT ACTIVITIES**	
Body scratching/rubbing	A subject uses its legs or a substrate (e.g., tree trunk) to rub part of its body
Vacuum*-*chewing	A subject chews with empty mouth
Head/body shaking	A subject vigorously shakes its head and/or body (not following wallowing or similar behavior)
Yawning	A subject performs deep, long inhalation with open mouth

For the reasons explained in the introduction, the behaviors possibly associated with transient anxiety (hereafter, target behaviors) in this study were *vacuum-chewing, yawning, head/body shaking*, and *body scratching/rubbing* (see Table 1 for a detailed description; Supplementary Videos 1–3). These behaviors are “events” [*sensu* (77)]; i.e., instantaneous patterns with no appreciable duration.

**Figure 1:**
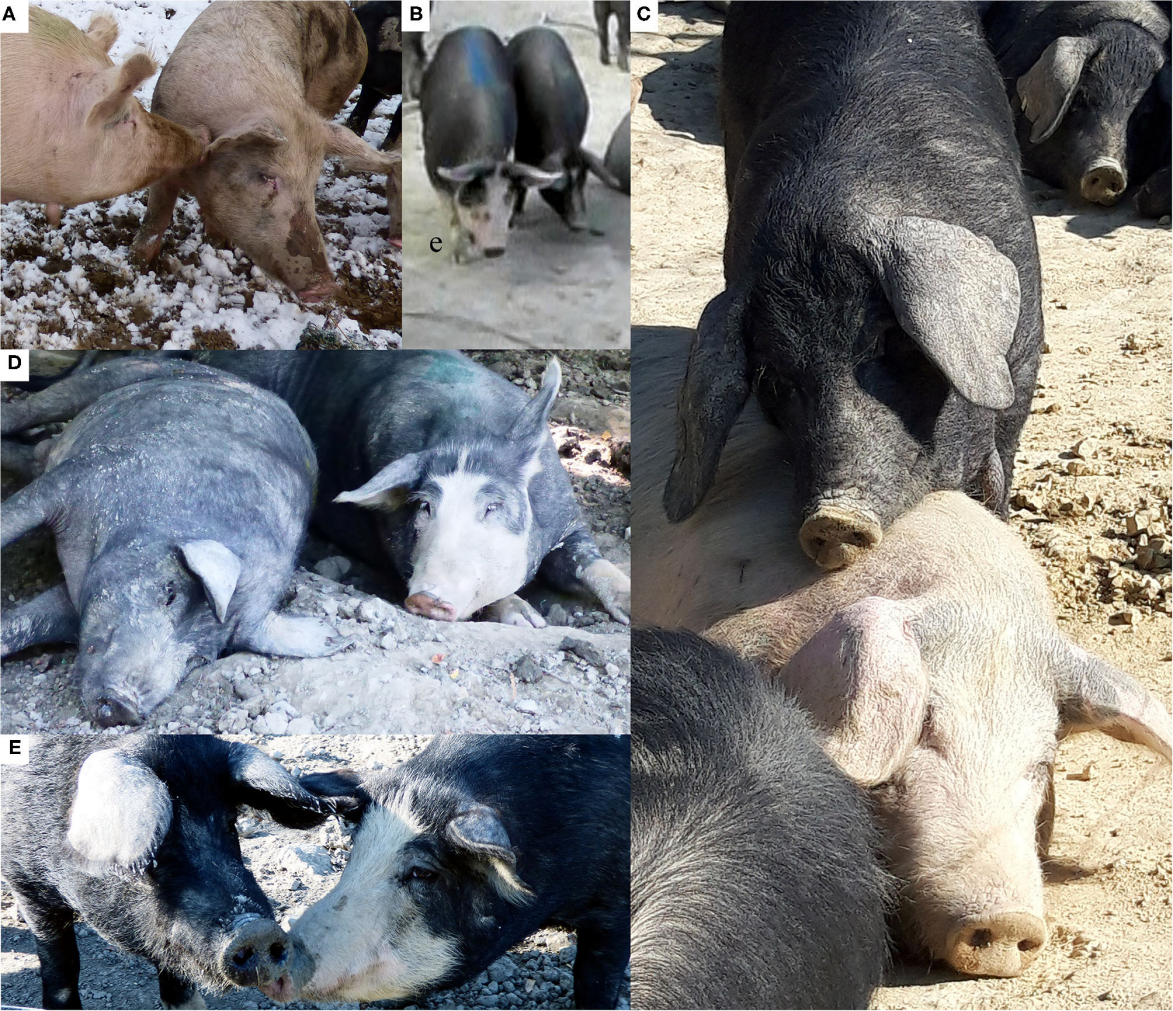
Examples of affinitive behaviors considered in this study. **(A)** nose-body contact; **(B)** social touching (body contact); **(C)** head-over; **(D)** rest-in-contact; **(E)** nosing body. Photo **(B)** was extracted from a video; Photos **(A,C–E)** by Ivan Norscia.

The social affiliation behaviors considered in this study are described in Table 1 and examples are shown in Figure 1. Inter-individual brief contact occurring by chance during foraging (over food items naturally available in the area) was not considered as affiliation and was excluded from the analyses. None of the affiliation behaviors considered prevented the animals from performing displacement activities.

The involvement of the subjects in aggressive encounters was coded as follows: (i) aggressor: the individual actively starting the agonistic interaction by attacking another individual (*via* at least one aggressive behavior described in Table 1); (ii) aggression recipient: any individual receiving the aggressive behavior or directly receiving physical contact from an aggressor during the aggressive event (e.g., in case of multiple aggressive events or aggression redirected by the recipient of a former aggression toward another subject); (iii) bystander: any individual present on the video during an aggressive event that received no physical contact with the opponents.

*Via* the all occurrences sampling method (77), all the behaviors detailed in Table 1 were collected from all individuals present in a video record when they were clearly visible, not sleeping and outside the periods when food was provided. This behavioral data was collected in 3-min blocks for each individual appearing in the video, falling under four conditions sharing the same or similar social context: before the stressful event (BA), 3 min after the stressful event in absence (AA) or in presence (AP) of affiliation, and in a matched-control condition (to assess behavioral baseline levels; MC). Following De Waal and Yoshihara (78), each matched control was paired with an observation around a conflict event and carried out during the next possible day in a similar context as the other conditions (similar weather and same time slot) but in the absence of perturbing events (aggression, sudden loud noises, food distribution, etc.) in the minutes right before the start of data collection in the conditions described above (we used 15-min as a buffer time window). In case of multiple aggressive events over a 3-min time slot, the time count started after the end of the last event. The double control (BA and MC)—compared with the other conditions—ensured that the observed behavioral variation could be directly linked to a stressful event. The three-min time window was selected for two reasons: (1) to ensure that the neuro-hormonal stress response linked to anxiety behavior was activated (79) and, (2) that the onset of the behavior directly followed the last aggressive event with no other perturbing event occurring in-between.

### Statistical Elaboration

#### Temporal Analyses

Owing to the non-normal distribution of the tested variables (Kolmogorov-Smirnov: N_subjects_ = 37–44, 0.001 < *p* ≤ 0.041) we applied different non-parametric tests at the individual animal level to check for changes in frequency of the target behaviors (80). In particular, we applied the Friedman's test for k ≥ 2 dependent samples *via* Monte Carlo randomization to carry out sequential analyses on the target behaviors (yawning, vacuum chewing, head/body shaking, and body scratching/rubbing) expressed by the study subjects (N_subjects_ = 44) across conditions (BA, AA, AP, MC). We applied the Dunn *post-hoc* test for pairwise comparisons, with the significance level of probability (fixed at 0.05) adjusted downward using the Bonferroni correction.

Next, we applied the same tests (Friedman's test and pairwise Bonferroni-Dunn *post-hoc* test) to check for variation in the levels of target behaviors across the 3 min following an aggressive event.

Subsequently, we applied the Wilcoxon's test for paired samples *via* Monte Carlo randomization to carry out a minute-by-minute analysis across the 3 min following a stressful event. *Via* this test we checked for differences in target behavior occurrence before (AA) and after affiliation (AP) in the first, second, and third minute after the aggressive event. The occurrence was calculated as the number of behaviors normalized over the events for each condition. The test was run on the individuals showing both conditions (presence/absence of affiliation; N_subjects_ = 37) in each of the 3 min. The Monte Carlo randomization (10,000 permutations) was applied to account for possible pseudo replication (same individual involved in different behaviors during different 1-min units) or pseudo-independency (more behaviors observed after the same aggressive event which may not be fully independent). Tests were run using SPSS 20.0.

#### Factors Possibly Linked to Anxiety Behavior Occurrence

We ran a Generalized Linear Mixed Model (GLMM) on the aggressive events (involving *N* = 74 individuals) to verify if different individual factors and the role played by the subject in the aggression could have an effect on the occurrence of anxiety related behavior. The occurrence of the target behavior (yawning, vacuum chewing, head/body shaking, and body scratching/rubbing) was entered as dependent, binary variable (coded as presence = 1, absence = 0). The fixed factors included in the full model were the following: (i) sex (factorial variable: M = males; F = females), (ii) age (numeric variables); (iii) breed (factorial variable: PB = Parma Black, L = Landrace, PIB = Piedmont Black; (iv) involvement of the subject in the aggression (A = aggressor, R = recipient, B = bystander). Because pigs were of mixed-breeds, the breed was assigned based on the mother's. The distinction of different categories depending of subject involvement was applied only for this test. The subject identity was included as random factor.

The models were fitted in R [(81); version 3.5.3] by using the function lmer of the R-package lme4 (82). As a first step it was verified if the full model significantly differed from the null model, including only the random factors (83). The likelihood ratio test (84) was used to test this significance (ANOVA with argument “Chisq”). Subsequently, by using the R-function “drop1,” the *p*-values for the individual predictors based on likelihood ratio tests between the full and the null model were calculated (85). As the response variable was binary, a binomial error distribution was used (link function: logit).

A multiple contrast package (multcomp) was used to perform all pairwise comparisons for each involvement category of significant fixed factors with the Tukey test (86). The Bonferroni-adjusted *p*-values were reported, along with estimate (Est), standard error (S.E.), and *z*-values.

### Ethics Statement

This research was purely observational and no animal manipulation was required during the study. Hence, no ethical approval was necessary according to current regulations.

## Results

### Yawning

**Figure 2:**
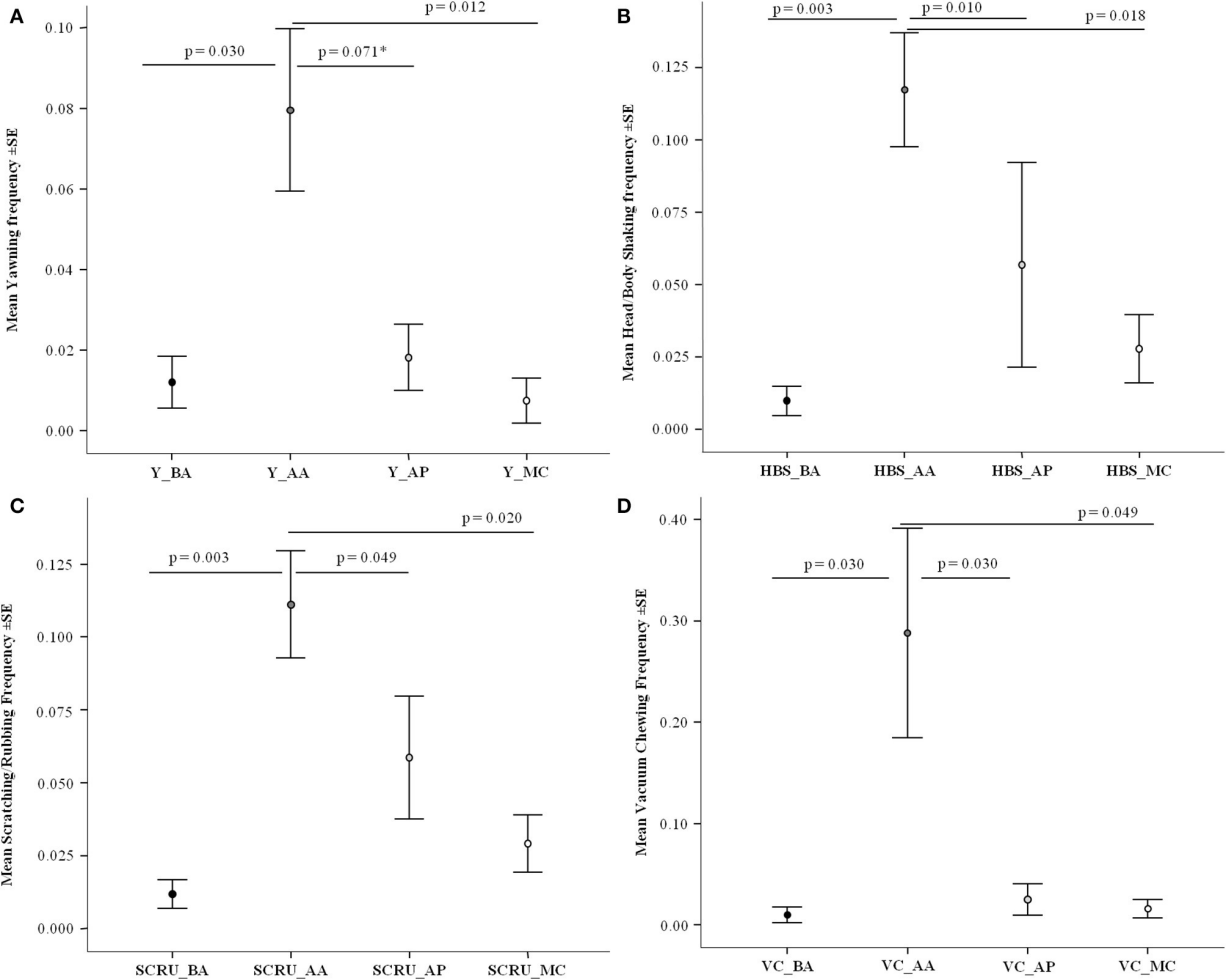
Anxiety-related behavior frequency (Y axis) as a function of four conditions (X axis): before aggression (BA), after aggression in absence (AA) and in presence of affiliation (AP), and matched control (no event; MC). In all cases **(A–D)** the difference across conditions was significant (Friedman's test: 26.749 ≤ χ^2^ ≤ 35.574, *p* < 0.001). Behavior considered and pairwise comparison results *via* Bonferroni-Dunn test: **(A)** Yawning (Y)—Significant differences: BA/AA (Q = −0.773, *p* = 0.030), MC/AA (Q = 0.852, *p* = 0.012); trend: AP/AA (Q = 0.693, *p* = 0.071); not significant: BA/AP (Q = −0.080, *p* = 1.000), MC/BA (Q = 0.080, *p* = 1.000), MC/AP (Q = 0.159, *p* = 1.000); **(B)** Head/body shaking (HBS)—Significant differences: BA/AA (Q = −0.955, *p* = 0.003), AP/AA (Q = 0.864, *p* = 0.010), MC/AA (Q = 0.818, *p* = 0.018); not significant: BA/AP (Q = −0.091, *p* = 1.000) BA/MC (Q = −0.136, *p* = 1.000), AP/MC (Q = −0.045, *p* = 1.000); **(C)** Body scratching/rubbing (SCRU)—Significant differences: BA/AA (Q = −0.966, *p* = 0.003), AP/AA (Q = 0.727, *p* = 0.049), and MC/AA (Q = 0.807, *p* = 0.020); not significant: BA/MC (Q = −0.159, *p* = 1.000), BA/AP (Q = −0.239, *p* = 1.000), MC/AP (Q = 0.080, *p* = 1.000); **(D)** Vacuum chewing (VC)—Significant differences: BA/AA (Q = −0.773, *p* = 0.030), AP/AA (Q = 0.773, *p* = 0.030), AA/MC (Q = 0.727, *p* = 0.049); not significant: BA/AP (Q = 0.000, *p* = 1.000); BA/MC (Q = −0.045, *p* = 1.000) AP/MC (Q = −0.045, *p* = 1.000). Vertical bars: Standard Error (SE) around the mean (circles). The asterisk marks the trend of significance (0.5 ≤ *p* ≤ 0.1).

The yawning frequencies were significantly different across the conditions considered: before the aggression (BA; Mean ± SD: 0.012 ± 0.032), after the aggression in the absence of affinitive contact (AA; 0.080 ± 0.134), after affinitive contact (AP; 0.018 ± 0.055), and in the Matched Control (MC; 0.010 ± 0.037) (Friedman test: *N* = 44, χ^2^ = 33.224, df = 3, *p* < 0.001). The pairwise comparisons (*via* Bonferroni-Dunn *post-hoc* test) revealed a significant difference between BA and AA (Q = −0.773, *p* = 0.030), and MC and AA (Q = 0.852, *p* = 0.012). A trend of significance was observed between AP and AA (Q = 0.693, *p* = 0.071). The difference was not significant between the other conditions (Figure 2A). Yawning significantly increased after exposure to an aggressive event and tended to decrease after affiliation, although not significantly.

### Head/Body Shaking

The frequencies of head-body shaking were significantly different across the conditions BA (Mean ± SD: 0.010 ± 0.034), AA (0.117 ± 0.130), AP (0.057 ± 0.235), and MC (0.028 ± 0.078) (Friedman test *via* Monte Carlo randomization: *N* = 44, χ^2^ = 34.030, df = 3, *p* < 0.001). The pairwise comparisons (*via* Bonferroni-Dunn *post-hoc* test) revealed a significant difference between BA and AA (Q = −0.955, *p* = 0.003), AP and AA (Q = 0.864, *p* = 0.010), and MC and AA (Q = 0.818, *p* = 0.018) but not between the other conditions (Figure 2B). Thus, the levels of head/body shaking significantly increased after an aggressive event and the frequency of such behavior was reduced to baseline levels after affiliation (Figure 2B, for example see Supplementary Video 4).

### Scratching/Rubbing

The frequencies of scratching/rubbing were significantly different across the conditions BA (Mean ± SD: 0.012 ± 0.032), AA (0.111 ± 0.122), AP (0.059 ± 0.139), and MC (0.029 ± 0.065) (Friedman test *via* Monte Carlo randomization: *N* = 44, χ^2^ = 26.749, df = 3, *p* < 0.001). The pairwise comparisons (*via* Bonferroni-Dunn *post-hoc* test) revealed a significant difference between BA and AA (Q = −0.966, *p* = 0.003), AP and AA (Q = 0.727, *p* = 0.049), and MC and AA (Q = 0.807, *p* = 0.020). No difference was found between the other conditions (Figure 2C). The levels of body scratching/rubbing significantly increased after an aggressive event and were reduced to baseline levels after affinitive contact (Figure 2C).

### Vacuum Chewing

The frequencies of vacuum chewing were significantly different across the conditions BA (Mean ± SD: 0.010 ± 0.052), AA (0.288 ± 0.686), AP (0.025 ± 0.102), and MC (0.016 ± 0.061) (Friedman test *via* Monte Carlo randomization: *N* = 44, χ^2^ = 35.574, df = 3, *p* < 0.001). The pairwise comparisons (*via* Bonferroni-Dunn *post-hoc* test) revealed a significant difference between BA and AA (Q = −0.773, *p* = 0.030), AP and AA (Q = 0.773, *p* = 0.030), and AA and MC (Q = 0.727, *p* = 0.049) but not between the other conditions (Figure 2D). Hence, the levels of vacuum chewing significantly increased after an aggressive event and were reduced to baseline levels after affiliation (Figure 2D).

### Minute by Minute Analysis

**Figure 3:**
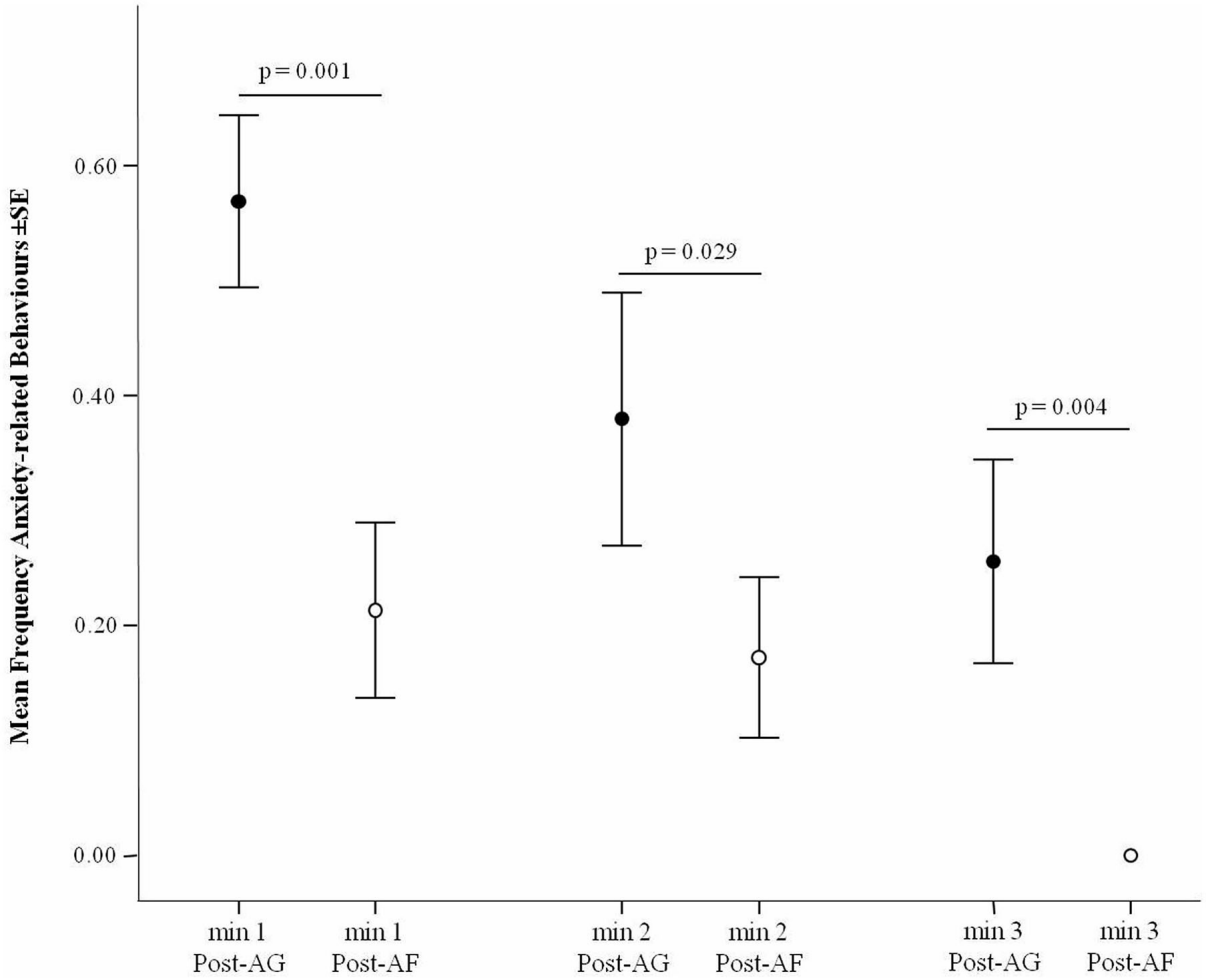
Frequency of anxiety-related behavior (Y axis) after aggression (Post-AG) and after affiliation (Post-AF) in each of the 3 min following the aggression (X axis). Vertical bars: Standard Error (SE) around the mean (circles).

The level of target behaviors significantly decreased over the 3 min following an aggressive event (Friedman test *via* Monte Carlo randomization: *N* = 37, χ^2^ = 28.777, df = 2, *p* < 0.001; Mean ± SD, min 1: 0.527 ± 0.385; min 2: 0.290 ± 0.424; min 3: 0.137 ± 0.316), and particularly between minute 1 to the other minutes (min 1 vs. min 2: Q = −0.662, *p* = 0.013; min 1 vs. min 3: Q = −1.122, *p* < 0.001) but not between minutes 2 and 3 (Q = −0.459, *p* = 0.144). The minute by minute analysis carried out across the 3 min following the stressful event revealed a significant difference in the levels of anxiety related behavior before (AE) and after affiliation (AA) in the first, second and third minute after the aggressive event (Wilcoxon's test *via* Monte Carlo randomization, *N* = 37; 1-min: T = 92.00, *p* = 0.001; 2-min: T = 53.00, *p* = 0.029; 3-min: T = 0.000, *p* = 0.004). In particular, in each given minute, the levels of anxiety related behavior were lower after affiliation than when affiliation did not occur (Figure 3).

### Factors Influencing Anxiety Behavior

**Figure 4:**
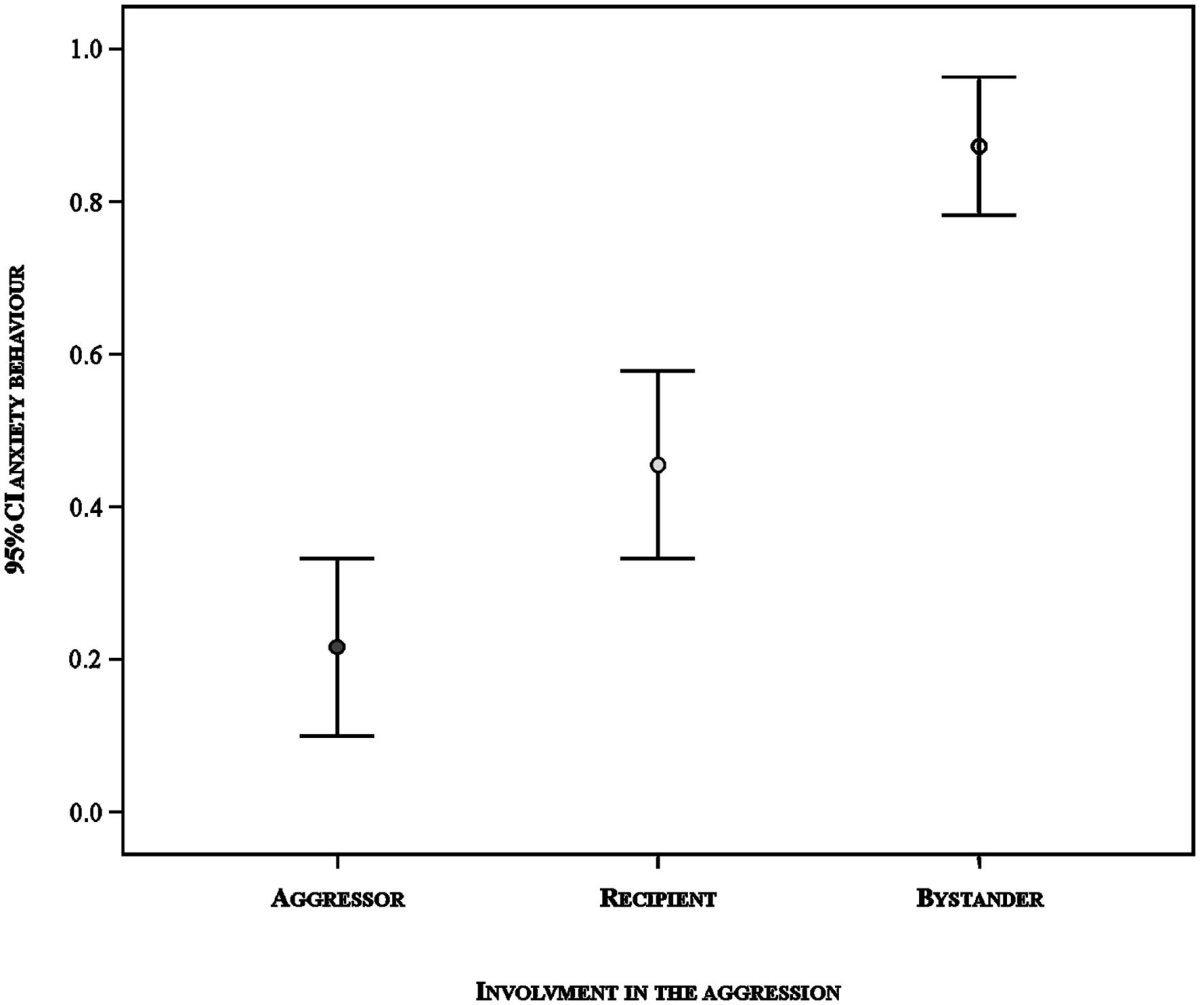
Likelihood of displacement activities in relation to the involvement of the subject in the aggression: aggressor, aggression's recipient and bystander. Vertical bars: 95% confidence interval around the mean (circles).

In the GLMM analysis, we found a significant difference between the full model versus a null model (likelihood ratio test: *N* = 172, χ^2^ = 58.384, df = 6, *p* < 0.001). Hence, we moved on with a drop1 procedure. The GLMM indicated a significant effect of the involvement of the subject in the agonistic event on the occurrence of anxiety behavior (Table 2). The other variables had no significant main effect. Specifically, the pairwise comparisons revealed that anxiety behavior was more likely to occur in the aggression recipients than in the aggressors (Tukey test: Est = −1.917; S.E. = 0.719, *z* = −2.667, *p* = 0.020) and in the bystanders than in the aggression recipients (Tukey test: Est = 2.397; S.E. = 0.630, *z* = 3.806, *p* < 0.001) and aggressors (Tukey test: Est = 4.313; S.E. = 0.946, *z* = 4.559, *p* < 0.001) (Figure 4).

**Table 2:** Results of the GLMM, including the following fixed factors: sex (factorial variable: M, males; F, females), age (scale variable), breed (factorial variable: PB, Parma Black; L, Large White; P, Piedmont Black), involvement of the subject in the aggression (R, recipient; A, aggressor; B, bystander).

**Fixed factors**	**Estimate**	***SE***	***z-value***	***P***
Intercept^a^	−1.364	1.286	*a*	*a*
Sex (M)^b^	−0.111	0.545	−0.204	0.838
Age	0.065	0.072	0.907	0.365
Breed (PB)^b^	0.141	0.807	0.175	0.861
Breed (L)^b^	0.931	0.765	1.172	0.241
Involvement (A)^b^	−1.916	0.719	−2.667	0.008
Involvement (B)^b^	2.396	0.630	3.806	<0.001

*The coded identity of the subject (nominal) was included as random factor. Full vs. null model: **χ**^2^ = 58.384, df = 6, p < 0.001*.

a *Not shown as not having a meaningful interpretation*.

b *Estimate ± SE refers to the difference of the response between the reported level of this categorical predictor and the reference category of the same predictor*.

## Discussion

The results of this study show for the first time that certain behaviors, part of the typical repertoire of *Sus scrofa*, can express anxiety, be buffered by social contacts, and possibly anticipate an imminent threat. Yawning, scratching/rubbing, head/body shaking, and vacuum chewing were found to express anxiety in our study subjects because such behaviors significantly increased after a stressful intra-group aggression (Prediction 1 confirmed; Figure 2). The frequencies of these behaviors decreased (or in case of yawning tended to decrease) to baseline levels—and did so faster in each of the 3 min following aggression—after that affinitive contacts occurred between group mates, which indicates the presence of a social buffering mechanism (Prediction 2 confirmed; Figures 2, 3). Finally, the occurrence of anxiety behavior varied depending on the different level of involvement of the subjects in aggression, thus suggesting that the behavioral expression of anxiety probably is not an all-or-nothing phenomenon but can depend on the level of threat that the subjects face (Prediction 3 confirmed; Table 2; Figure 4). Intriguingly, our results show that after aggression, bystanders showed higher frequencies of anxiety related behavior than the individuals directly involved in the aggression (Figure 4), which suggests the ability of domestic pigs to anticipate, at least in the short term, the potential threat (aggression risk) that a tense situation bears. Here below we discuss these results in detail.

### Anxiety Related Behavior

The fact that scratching/rubbing, head/body shaking, yawning, and vacuum chewing increased after a stressful event is in line with most of the ethological and physiological findings on other species. Self-directed behavior, including scratching, has been found to increase in tense situations in humans (4, 36) and several other mammalian species [e.g., chimpanzees, *Pan troglodytes* (39); long-tailed macaque, *Macaca fascicularis* (87); Olive baboons, *Papio Anubis* (88); brown lemur, *Eulemur rufus*x*collaris* (25); ring-tailed lemur, *Lemur catta* (27); dogs (89)]. Shaking—which can include different body parts depending on the species—or trembling has been reported as a sign of anxiety increase in humans (35) and dogs (90), although it was not found to increase after stressful events in horses (91). Yawning has been associated to anxiety in humans (37) and other mammalian species [South American sea lions, *Otaria flavescens* (44); Verreaux' sifaka, *Propithecus verreauxi* and ring-tailed lemurs (92); rats (45)]. Vacuum chewing can increase or be associated with stressful events in horses (41, 42, 91), although in horses this variation is not necessarily associated with stress-related variation of heart rate parameters (91). This result is possibly due to the fact that vacuum chewing in horses may be associated not just with stress-related anxiety but also with positive emotions (91, 93). Finally, vacuum chewing and yawning have been reported in pigs as stereotypic, abnormal behavior associated with chronic stress (47, 94–97). Our results show that the behaviors considered in this study as part of the typical repertoire of the species, can indicate a transient anxiety increase in ecologically sound contexts and in the absence of sources of chronic stress, such as confinement and overcrowding.

Physiological studies, combined with ethological observation and pharmacological manipulation, have provided insights on the mechanisms underlying anxiety behaviors. In particular, such studies have shown the link between the stress response mediated by glucocorticoids and scratching in mammals [dogs (89); primates (4, 49, 50, 87)]. An analogous link has been demonstrated in pigs for stereotypic vacuum chewing (47), indirectly suggested in dogs for body shaking (40), and described in rats (45) and hypothesized in humans for yawning (46, 98). Our results suggest that a similar association might be present in domestic pigs for the anxiety related behaviors considered in this study. Further studies linking hormonal and behavioral variations are necessary to confirm this hypothesis.

### Social Buffering of Anxiety Behavior

As a second point, our results show that the social buffering of anxiety is present in domestic pigs living in naturalistic conditions because social affiliation with group mates decreased the level of anxiety related behaviors, restoring baseline levels. For yawning a decrease trend was observed after affiliation. This result might indicate that social buffering works also for yawning, but more data are necessary to confirm this. Alternatively, this result may be due to the fact that yawning is associated not just with an increase in anxiety levels, but more generally to physiological and behavioral changes, such as those occurring over the sleep-wake cycle or during the transition between stress arousal and relaxation (37, 92). Hence, yawning can be expressed regardless of the direction of transition (e.g., from relaxation to anxiety or from anxiety to relaxation). Affinitive contacts can activate hormonal responses leading to calm restoration after anxiety arousal (65–67). Thus, in our case, the relaxing effect of social affiliation may have buffered a decrease in yawning.

With regards to the other behaviors considered in this study (head/body shaking, vacuum chewing, and scratching/rubbing), our results show that their frequency decreased to baseline levels following affinitive contacts between group mates.

Our results show that the levels of anxiety related behavior decreased over the 3 min following the aggressive event with a peak occurring in the first minute. The minute-by-minute analysis showed that the decrease of anxiety behavior was not only due a physiological decline occurring from the time aggression elapsed. In each of the three consecutive minutes following the aggressive event, the frequency of anxiety behavior was significantly lower in the presence of, rather than in the absence of affiliation (Figure 3). Hence, affiliative behavior boosted the reduction of anxiety behavior in the domestic pigs under study from the first minute. This result is consistent with previous research on pigs showing that they are sensitive to the social presence or vocal support of conspecifics (99–101) and that the simple proximity with group mates can reduce stress (57). More in general, our results confirm that positive social interactions can be crucial for the health and well-being of domestic animals (102).

The reduction of anxiety behavior after affiliation has been observed in different social mammals, and particularly primates. For example, self-directed behavior can decrease after grooming in the ring-tailed lemur (27) and after playful social contacts in the common marmosets [*Callithrix jacchus* (59)], a New World monkey species. In humans, social affiliation can lead to anxiety reduction and better performance (103). Conciliatory contacts between former opponents were found to work in reducing anxiety behavior in brown lemurs (25), several Old World monkey species [olive baboons, *Papio anubis* (61); macaques, *Macaca* spp. (60, 62, 104, 105)], wallabies (43), and domestic goats (106). Affinitive contact with a group member different from the aggressor was found to reduce post-conflict anxiety behavior in the victim of aggression in chimpanzees [(63); but see (64)] and bonobos (107, 108).

The effectiveness of social interactions in reducing anxiety behavior in domestic pigs probably reflects the importance of social relationships in this species. As a matter of fact, immature individuals of this species start establishing inter-individual relationships *via* play first with littermates and later also with other conspecifics (109–111). Socialization in early life also provides individuals with greater confidence and agonistic skills, which help to reduce the negative effects of aggression (112). In the wild counterpart, long-term relationships persist between adult females (113). Affiliation can be promoted by an increase of stress-related anxiety (possibly mediated by oxytocin) and produce a calming effect (114, 115). From an evolutionary perspective, it may have been favored to avoid the disruption of social groups. Group disruption clearly has a negative impact on the chances of survival for individuals living in social settings, considering that inter-individual interactions are crucial to obtain cooperation from others, provide protection from environmental threats, and enhance better recovery from aversive experiences (32). The importance of social interactions, not limited to the domesticated form, may not be strictly connected to domestication. The evolutionary history of *Sus scrofa* started long before (around 20 million years ago) and has lasted much longer than its domestication process, which began around 10,000 years ago (116, 117). It is likely that certain features of the biology of *Sus scrofa* that are found in both the domestic and the wild form (such as social traits related to survival increase) have emerged and have been favored by natural selection before artificial selection (operated by humans during domestication) came into play. Our results confirm the importance of inter-individual affiliation and social buffering in domestic pigs, as a mean to accelerate a decrease in anxiety and to quickly restore group homeostasis and individual welfare.

### Anxiety Behavior and Level of Threat

Our results indicate no significant effect of age, breed, and sex on the occurrence of anxiety related behavior after aggression. The fact that all individuals were adult, mixed breed, and that males were castrated may have dampened possible differences in the behavioral expression of anxiety. However, our findings indicate that the behavioral expression of anxiety in domestic pigs can vary depending on the level of threat, real, or potential, that the individuals face. In the study subjects, anxiety behavior was more expressed in the recipients of aggression, physically involved in the aggression in a defensive way, than in aggressors, which played an active role in attacking other individuals (Figure 4). This is in line with previous literature [but for example see (43, 118)] which tends to indicate, on a general scale, that submissive subjects experience more stress related anxiety than aggressive individuals [rats (26); ring-tailed lemurs (27); olive baboons (28, 119); humans (114, 120)]. On a smaller scale, when considering single aggressive events, leading a fight might help release anxiety in domestic pigs in the same way as in primates (24). This anxiety relieving effect might possibly be due to the divergent effect of testosterone and cortisol, which seems to be involved in aggressive approach and fearful withdrawal, respectively (121).

Our findings also indicate that bystanders also expressed anxiety behavior after a conflict. The fact that anxiety behavior can increase in uninvolved individuals has been previously reported in primates, for example in the Old World monkeys *Papio hamadryas* (29, 30) and *Mandrillo sphinx* (122) in which displacement activities increased in the individuals that were only witnessing a fight and not taking part in it. In canids and primates, bystanders can be implicated in post-conflict dynamics in various ways (e.g., by interacting aggressively or in an affinitive way with aggressor and/or aggressor's recipient) to restore social cohesiveness, reduce anxiety in others, or for self-protection [e.g., (63, 107, 108, 123–129)]. This implication of bystanders in conflicts in different species suggests that the spread of social tension following aggression may be common in many social mammals (122).

In our study group pigs that witnessed an aggression did not just show an increase in anxiety behaviors; they also showed the highest level of increase compared to those individuals directly involved in aggression. This result draws attention to at least two aspects that may deserve future detailed investigation.

The first is that anxiety and fear are two intertwined but different emotional states, which in both humans and rodents can involve overlapping but also different areas of the brain (130, 131). Fear is a response to short, present, aversive cues leading, for example, to fleeing behavior (130, 132). Anxiety on the other hand is a psychological, physiological, and behavioral state induced in animals by an imminent or potential threat (15), leading to displacement activities (4). Hence, recipients of aggression, when under attack, may flee (for a present aversive cue) whereas bystanders may stay and experience anxiety arousal linked to the imminent, potential threat posed by the intra-group aggression involving others. In this study, we could not investigate fear responses, as the pigs experienced very low disturbance and very rarely faced actual fearful situations. Redirected aggression from former opponents (especially recipients) to bystanders has been found to occur after conflicts in mammals, for example in primates (133, 134) and wolves (125, 135, 136). Thus, the threat of aggression can be related to the possibility that conflict involving other subjects triggers an aggressive chain reaction hitting other group members. In the pigs of our study group, the likely different emotional states experienced by the actual recipients of aggression (victims) and potential recipients (bystanders) may account for the differences in the frequencies of anxiety behavior observed in these two categories. Further studies on pig emotional states and more generally on animal emotions are necessary to fully discern between the behavioral markers of fear and anxiety. The second aspect concerns the possible threat anticipation in domestic pigs. The additional anxiety behavior shown by bystanders compared to involved subjects points toward the possibility that pigs are able to emotionally respond to the imminent threat posed by an aggression. This observation is in line with previous findings showing that *Sus scrofa* possess short-term anticipatory skills, detected in other domains. Specifically, pigs were found to reduce aggression and increase play in anticipation of enrichment (137) and increase their activity levels in anticipation of food (138). Moreover, pigs seem to be able to share the physiological state of others [yawn contagion and emotional contagion (139, 140)]. Moreover, pigs seem to be able to not only mimic the emotional behavior of conspecifics and share the emotional states of others, but also synchronize their emotions with pigs that are responding emotionally in anticipation of future events (57, 140, 141).

In conclusion, this study provides the first eco-ethological insight on how domestic pigs living in a natural habitat can behaviorally express non-pathological anxiety—also in anticipation of potential imminent threats—and how such anxiety can be socially buffered. These aspects are worth considering for extensive farming management under a welfare perspective. It is important to take into account not only anxiety behavior but also whether it is ensured that the animals are provided with enough space to avoid situations perceived as risky, allowing them to properly engage in positive social interactions, useful for buffering negative emotional states. Finally, owing to the ecological context in which the animals lived and the absence of abnormal of stereotypic behaviors, this study can also provide the ground work for a better understanding of the social traits that are hard wired in the species' biology and that may be the result of evolutionary convergences between domestic pigs and other highly cognitive mammals, such as human and non-human primates.

## Data Availability Statement

The raw data supporting the conclusions of this article will be made available by the authors, without undue reservation.

## Ethics Statement

Ethical review and approval was not required for the animal study because it consisted in a purely observational study with no manipulation.

## Author Contributions

EC: data collection. IN and GC: conceptualization, methodology, writing—original draft preparation, and writing—review and editing. IN, GC, and EC: formal analysis and investigation. All authors: contributed to the article and approved the submitted version.

## Conflict of Interest

The authors declare that the research was conducted in the absence of any commercial or financial relationships that could be construed as a potential conflict of interest.
