# Supplementary material for: Anxiety Behavior in Pigs (Sus scrofa) Decreases Through Affiliation and May Anticipate Threat
Source: Front Vet Sci. 2021 Feb 16;8:630164. doi: 10.3389/fvets.2021.630164 (PMC7921160; doi:10.3389/fvets.2021.630164)
Supplement: Supplementary file 5 [file Table_1.DOCX]

**Video legends**

**Video 1**

Three behavioral patterns are are shown here: vacuum chewing (repeatedly done by the large white pig on the right), yawning (displayed by the Piedmont Black pig on the left) and head-shaking (performed by the large white pig entering from left at second 6).

**Video 2**

An individual rubbing its head/neck against a tree trunk

**Video 3**

An individual self-scratching

**Video 4**

Aggressive mount between two males (Parma Black over Large White subject) and subsequent head-shaking in a bystander (Parma Black).
